# CT-based radiographic measurements and effectiveness estimates of full-endoscopic surgery in thoracic myelopathy caused by ossification of ligamentum flavum

**DOI:** 10.1186/s12893-023-01989-6

**Published:** 2023-04-11

**Authors:** Jia-lin He, Qian Du, Wan-dong Hu, Zhi-jun Xin, Xin-xin Shao, Wen-bo Liao

**Affiliations:** 1 The Second Affiliated Hospital of Zunyi Medical University, Zunyi, 563006 Guizhou China; 2 Guizhou Aerospace Hospital, Zunyi, Guizhou, 563099 China; 3 Affiliated Hospital of Zunyi Medical University, Zunyi, 563000 Guizhou China

**Keywords:** Ossification of ligamentum flavum, Thoracic myelopathy, Computed tomography, Minimally invasive spine surgery.

## Abstract

**Background:**

Evaluate the effectiveness of posterior percutaneous full-endoscopic technique for patients with thoracic myelopathy caused by ossification of ligamentum flavum (TOLF).

**Methods:**

A prospective study was conducted for 16 patients with TOLF, who were treated with posterior endoscopic technique from 2017 to 2019. The sagittal and cross-sectional CT images are used to measure the area of ossified ligamentum and evaluate the decompression of surgery, respectively. The effectiveness was evaluated with visual analog scale (VAS), modified Japanese Orthopedic Association scale (mJOA), The Oswestry Disability Index (ODI), and Macnab efficacy evaluation.

**Results:**

The average area of TOLF on sagittal and cross-sectional CT images in the 16 patients was (116.62 ± 32.72) mm^2^ and (141.59 ± 27.25) mm^2^ preoperatively, (15.99 ± 12.54) mm^2^ and (11.72 ± 8.64) mm^2^ at 3 days after the operation, (16.78 ± 11.49) mm^2^ and (10.82 ± 7.57) mm^2^ postoperative 1 year, respectively. The invasive proportion of spinal canal at preoperative sagittal and cross-sectional CT images was (48.10 ± 10.04) % and (57.58 ± 11.37) %, which decreased to (6.83 ± 4.48) % and (4.40 ± 3.01) % at the final follow-up. The average score of mJOA, VAS and ODI improved. The excellent and good rate was 87.50% according to Macnab evaluation. Compared with preoperative, differences in areas of TOLF, proportions of spinal canal, and clinical assessments of postoperative 3 days and 1 year were all statistically significant. Two cases of dural tear were observed.

**Conclusion:**

Endoscopic surgery has a good clinical effect on TOLF, which has the advantage of less trauma to the paraspinal muscles and no impact on the spinal structure. The CT-based radiographic measurements can quantitatively evaluate the degree of spinal canal stenosis in TOLF.

## Background

Ossification of ligamentum flavum (OLF) is common in the East Asian population. The fibrous tissue in the ligamentum flavum transforms into bone tissue and proliferates, which occupies the spinal canal and compresses the spinal cord. Then the compression-related symptoms will be presented. OLF may occur at any spinal segment, and the most common level was at the thoracic ones, especially the lower segments (T9 ~ T12) [1–3].

As one of the major causes of thoracic myelopathy (TM), thoracic myelopathy caused by OLF (TOLF) is most common in individuals 45 ~ 65 years old and often progresses secretly without obvious clinical symptoms in early stage [4–6]. In the middle and late stages of TOLF, the ossified ligamentum flavum compresses the spinal cord, which can cause sensory and/or motor disorders of trunk and limbs, and patients often have severe conditions once they have apparent symptoms. If spinal cord nerve decompression is not performed in a timely manner, the patients may exhibit permanent loss of nerve function or even paralysis.

At present, surgery is the only treatment for TM caused by OLF that is considered to be definitely effective. The traditional ways of operation most commonly involve laminectomy, as well as semi-laminectomy, laminoplasty and intervertebral foramen surgery [7]. By removing the ossified ligamentum flavum and/or expanding the spinal canal to decompress the spinal cord, the nerve could be saved. Although the clinical effect of traditional treatments for thoracic ligamentum flavum ossification were clear, it still has many disadvantages, such as substantial trauma and a high rate of complications (34%~76.7%) [8–13], which often lead to additional pain and financial burden in patients and their families. In recent years, the endoscopic technique has been used for the treatment of ossification of the thoracic ligamentum flavum [14–17]. Compared with traditional open surgery, endoscopic surgery has many advantages, such as minor trauma, lower costs and less impact on spinal stability. However, it is not clear whether endoscopic surgery can achieve full decompression in the spinal canal. To further determine whether spinal endoscopic surgery is clinically efficacious in treating TOLF and whether the degree of spinal canal decompression achieved is adequate, we evaluated the spinal canal decompression degree and clinical efficacy of 16 patients with TM caused by OLF before, 3 days and 1 year after translaminar osseous channel–assisted full-Endoscopic flavectomy.

## Methods

### General information

A total of 16 patients with TM caused by OLF were selected as subjects according to the inclusion and exclusion criteria.

The inclusion criteria were as follows: having received a clinical diagnosis of single-segment OLF, having received indications for surgery, having complete clinical data and imaging examination data.

The exclusion criteria were as follows: severe internal medical diseases such as cardiovascular and cerebrovascular diseases, respiratory diseases, and dysfunction of the liver or kidney; other severe degenerative diseases; cognitive impairment or mental illness; and incomplete clinical and radiological data.

General data of patients were collected: age, gender, diseased region, duration of disease, clinical manifestations etc. The study was approved by the Ethics Committee of our hospital and all patients had been informed of the study details and signed informed consent approvals.

### Perioperative conditions

Perioperative conditions of patients were recorded: operative time, the volume of intraoperative bleeding, the length of hospital stay, wound healing, and postoperative complications.

### Surgical methods

**Fig. 1:**
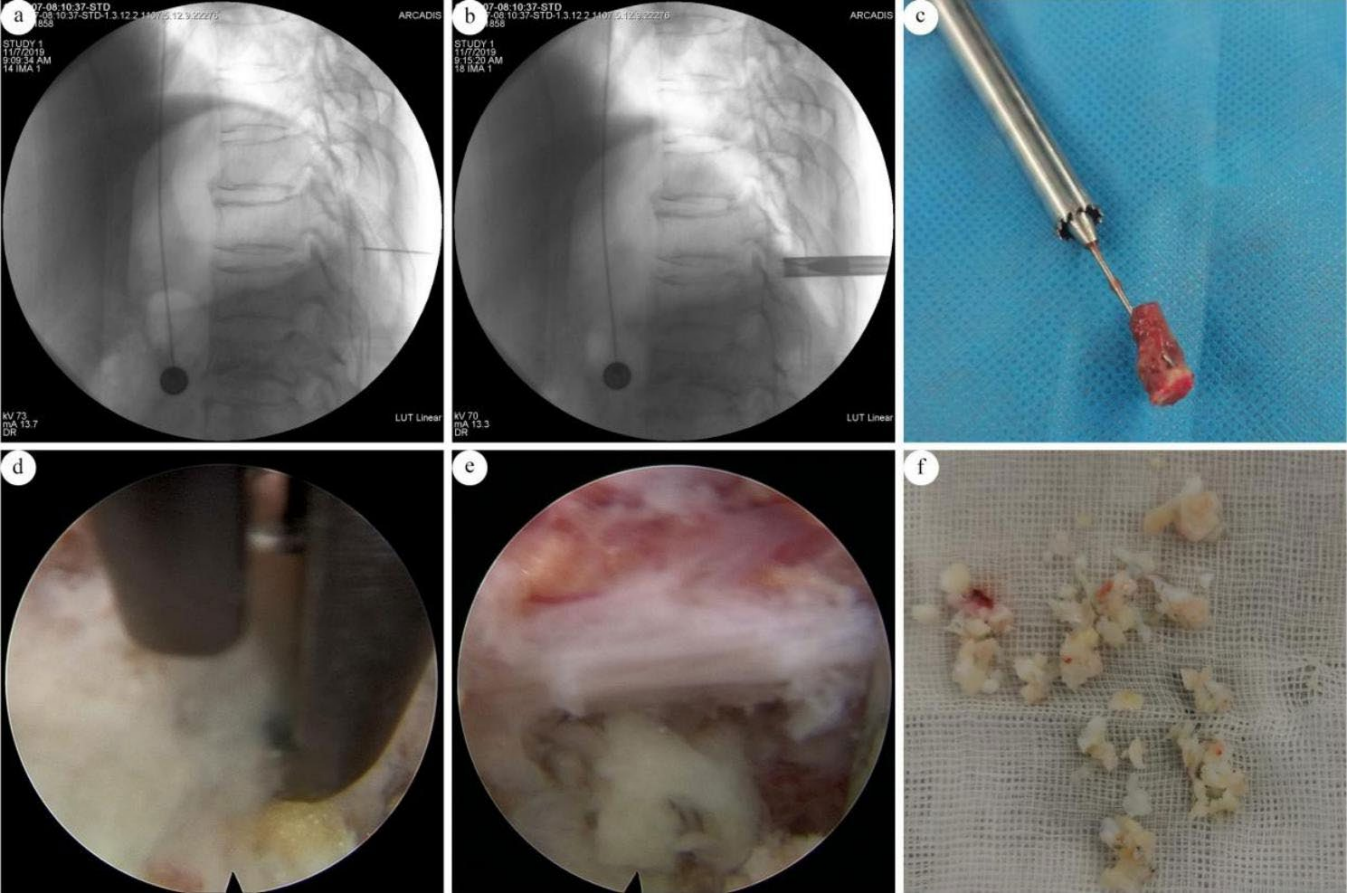
(a) A Kirschner needle was located on the posterior osseous lamina behind of the lesion segment; (b) A ring saw was used to remove a piece of the lamina to make a tunnel; (c) a piece of lamina was removed to get adequate operational space; (d) The ossified ligamentum flavum was resected by endoscopic rongeur; (e) The space around the dural sac was wide and decompression was adequate; (f) Collected samples of ossified ligamentum flavum

All patients were anesthetized with tracheal intubation. After anesthesia, the patient was placed in a prone position. Preoperatively, a C-arm X-ray machine was used for visualization, and the projection of the diseased vertebrae and the adjacent upper and lower vertebrae were marked on the body surface. Routine disinfection was performed, and aseptic towels were spread. A puncture was made by a Kirschner needle at the point 1 to 2 cm outside the posterior median line to locate the lateral lamina behind the lesion segment. With the guidance of C-arm visualization, the Kirschner needle was confirmed to be located on the posterior osseous wall of the lamina, and its axis was extended to the ossified ligamentum flavum (Fig. 1a). An incision measuring approximately 7 mm was made on the skin, the Kirschner needle was inserted into the extension rod, and the working catheter was inserted into the posterior wall of the spinal canal. A ring saw (Fig. 1c) was used to remove a piece of the lamina to make a tunnel (Fig. 1b), the guide rod, laminar bone piece and ring saw were removed (Fig. 1c) together, and the endoscopic system was placed. The ossified ligamentum flavum was then resected by endoscopic rongeur to decompress the spinal cord and nerve (Fig. 1d & e). To explore the dural sac, radiofrequency ablation of surrounding soft tissues was performed. The catheter was removed after saline irrigation, and the skin was sutured. Samples of the ossified ligamentum flavum were collected for pathological examination (Fig. 1f). The operative time and the amount of bleeding were recorded, and the changes in symptoms and signs were observed after the operation.

### Imaging measurements

A thoracic computed tomography (CT) scan was performed before, 3 days and 1 year after the operation. SURGIMAP software (Nemaris Inc, New York) was used to measure the area and proportion of the OLF and the invasive proportion of spinal canals.

**Fig. 2:**
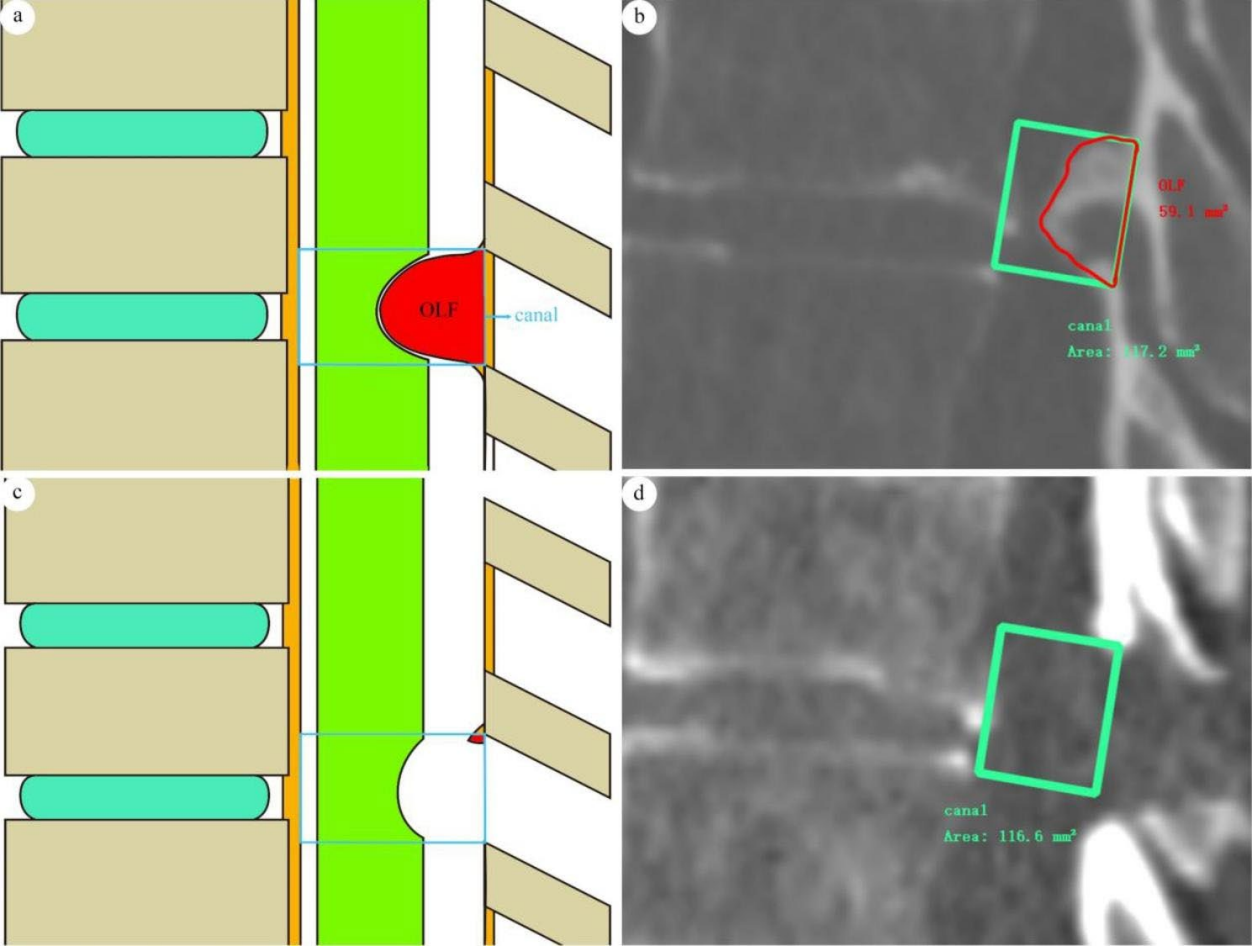
(a) diagram of preoperational sagittal measurements; (b) An actual preoperational measurement, the sagittal OLF area was 59.1 mm^2^, the sagittal canal area was 117.2 mm^2^, and the saggital invasive proportion was 50.43%; (c) diagram of postoperational sagittal measurements; (d) An actual postoperational measurement, the area of OLF was 0 mm^2^, the area of canal was 117.2 mm^2^, and the invasive proportion was 0%

The sagittal area of OLF on the CT scans (As) was measured as follows:

For the preoperative measurements, the sagittal CT images with the deepest OLF area were selected. Mark the upper and lower ends of the ossified ligamentum flavum (the junction of OLF and the upper/lower lamina), draw two lines from the upper and lower ends at the end points in the posterior wall of spinal canal, make the lines perpendicular to posterior wall of spinal canal, and the anterior wall of the spinal canal was intersected by the lines at two points. Two anterior points and two posterior points make up a rectangular region; that is, the sagittal area of the canal. The high-density region in the sagittal spinal canal area was outlined by using SURGIMAP, and the area of ossified ligamentum flavum was measured (Fig. 2a & b).

For the postoperative measurements, the same level of the sagittal CT scans was selected, and the same rectangular region was selected as the sagittal spinal canal area. The residual OLF area was measured (Fig. 2c & d). The proportion of OLF on the sagittal CT scans was measured as the ratio of the ossified ligamentum flavum area to the spinal canal area.

**Fig. 3:**
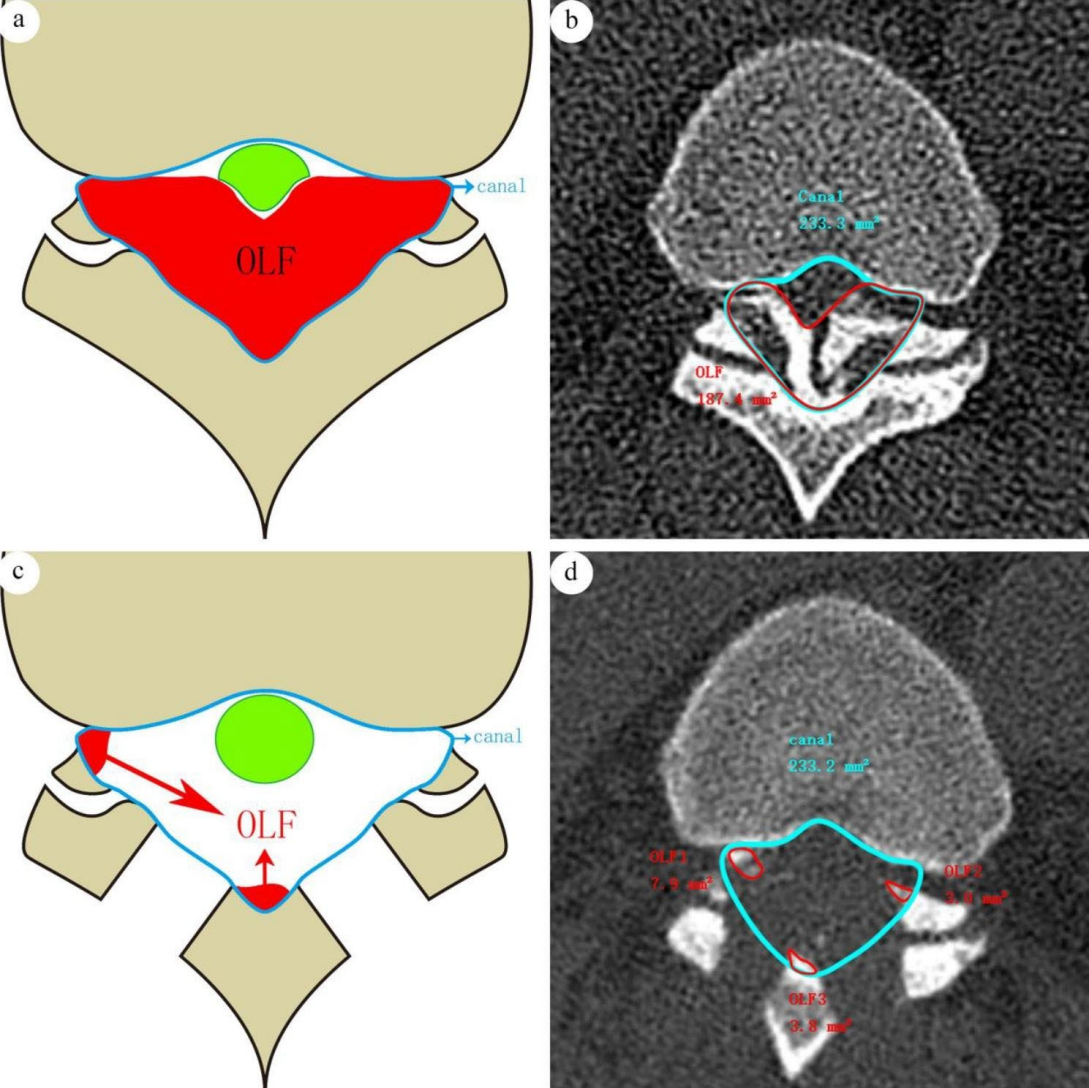
(a) diagram of preoperational cross-sectional measurements; (b) An actual preoperational cross-sectional measurement, the area of OLF was 187.4 mm^2^, the area of canal was 233.3 mm^2^, and the invasive proportion was 80.33%; (c) diagram of postoperational cross-sectional measurements; (d) An actual postoperational cross-sectional measurement, the area of OLF was 14.7 mm^2^, the area of canal was 233.2 mm^2^, and the invasive proportion was 6.30%

The cross-sectional area of OLF on the CT scans was measured as follows:

For the preoperative measurements, the scan with the deepest OLF was selected, and SURGIMAP software was used to outline the spinal canal wall contour, including the posterior wall of the vertebral body, the lamina and the articular process joint. The surrounding area was a cross-sectional canal area. The high-density region in the area of the cross-sectional canal, as the cross-sectional OLF area, was outlined and measured (Fig. 3a & b).

For the postoperative measurements, the CT scan of the same location as preoperative was selected, the same spinal canal area was selected as preoperative, and the contour of the area was outlined. The residual OLF area was measured with the same method (Fig. 3c & d). The cross-sectional proportion of OLF was measured as the ratio of the cross-sectonal OLF area to the cross-sectional canal area.

### Clinical assessment

The clinical symptoms were evaluated by the modified Japanese Association of Orthopedics scale (mJOA), visual analog scale for pain (VAS), Oswestry lumbar dysfunction index(ODI) before the operation, 3 days after the operation and 1 year after the operation. And Macnab evaluation was performed postoperative 1 year. The Hirabayashi recovery rate of all patients was calculated using the preoperative and final follow-up mJOA scores [18].

### Statistical analyses

Statistical analyses were performed using SPSS 22.0 (SPSS Inc., Chicago, IL) and continuous data are presented as the mean ± standard deviation (SD). The one-way analysis of variance was used to evaluate whether there were differences between preoperative, postoperative, and final parameters, and *P* values < 0.05 were considered statistically significant.

## Results

### General information

This study included 16 patients. The T11/12 segments were affected in 6 patients, the T10/11 segments were affected in 6 patients, and the T9/10 segments were affected in four patients. All cases involved the lower thoracic vertebrae. Of these patients, 7 were male and 9 were female. Patients ranged in age from 46 to 68 years, with an average age of 54.31 years. The duration of the disease ranged from 1 to 24 months, with an average duration of 7.1 months. The clinical manifestations of the disease were sensory disorders in 14 patients, lower limb muscle loss in 9 patients, sphincter dysfunction in 0 patients, chest and waist band sensation in 1 patient, intermittent claudication in 6 patients, lumbar and leg pain in 11 patients.

### Perioperative conditions

Sixteen patients successfully completed full endoscopic surgery. The operative time was 91–260 min, with an average of (137.81 ± 41.98) min; the volume of intraoperative bleeding was 10 ~ 80 ml, with an average of (25.63 ± 17.689) ml; the length of the hospital stay was 3 ~ 9 days, with an average of (5.06 ± 1.78) days. The wounds healed well, and there were no postoperative infections, no perioperative deaths, and two cases of intraoperative dural tears. However, no cases of postoperative CSF leakage were observed. There were no complications, such as nerve injury or incision infection.

### Comparison of OLF area of CT images before and after surgery

The average area of TOLF on preoperative sagittal CT images in the 16 patients was (116.62 ± 32.72) mm^2^, (15.99 ± 12.54) mm^2^ at 3 days after the operation and (16.78 ± 11.49) mm^2^ at 1 year postoperatively. The average area of TOLF on preoperative cross-sectional CT images was (141.59 ± 27.25) mm^2^, (11.72 ± 8.64) mm^2^ at 3 days after the operation and (10.82 ± 7.57) mm^2^ at postoperative 1 year. The invasive proportion of spinal canal at preoperative sagittal and cross-sectional CT images was (48.10 ± 10.04) % and (57.58 ± 11.37) %, which decreased to (6.83 ± 4.48) % and (4.40 ± 3.01) % at the final follow-up. The percentage of ossification area in sagittal position and transverse position at 3 days and 1 year after surgery was significantly smaller than that before surgery (*P*＜0.05), and there was no significant change one year after operation compared with three days after the operation (*P*＜0.05). The results of radiographic measurements are shown in Table 1.

**Table 1:** The radiographic measurements in 16 patients at different time points

Time	Sagittal OLF area (mm^2^)	Sagittal OLF Proportion (%)	Cross OLF area (mm^2^)	Cross OLF Proportion (%)
Preoperatively	116.62 ± 32.72	48.10 ± 10.04	141.59 ± 27.25	57.58 ± 11.37
3 days after the operation	15.99 ± 12.54*	6.46 ± 486*	11.72 ± 8.64*	4.76 ± 3.45*
1 year after the operation	16.78 ± 11.49*	6.83 ± 4.48*	10.82 ± 7.57*	4.40 ± 3.01*

*: Compared with the “Preoperatively” data, the differences were statistically significant (*P* < 0.05). Continuous data were presented as the mean ± standard deviation (SD).

### Comparison of clinical symptom scores before and after surgery

As shown in Table 2, postoperative mJOA score and ODI score were significantly higher than that before surgery. Postoperative VAS score was lower than that before surgery, and the differences were statistically significant (*P*＜0.05).

**Table 2:** The evaluation of the mJOA、VAS、ODI scores of 16 patients at different time points

Time	mJOA	VAS	ODI (%)
Preoperatively	3.50 ± 1.10	5.94 ± 1.18	45.63 ± 6.90
3 days after the operation	6.19 ± 0.91*	3.38 ± 1.50*	62.13 ± 11.25*
1 year after the operation	9.19 ± 1.38*^#^	1.19 ± 1.05*^#^	89.75 ± 5.16*^#^

*: Compared with the “Preoperatively” data, the differences were statistically significant (*P* < 0.05) #: Compared with the “3 days after the operation” data, the differences were statistically significant (*P* < 0.05). Continuous data were presented as the mean ± standard deviation (SD).

### Recovery rate

The overall Hirabayashi recovery rate was 73.96% ± 16.58% by calculating MJOA scores before operation and at the last follow-up. According to the Macnab’s evaluation, the recovery status of the 16 patients one year after the operation was excellent in 9 patients, good in 5 patients, and fair in 2 patients; the excellent and good rate was 87.50%.

## Discussion

The spinal endoscopic system was first used to achieve endoscopic surgery in a case of thoracic myelopathy caused by the ossified ligamentum flavum by Ikuta in 2011 [14]. In recent years, some surgeons [15, 17] have reported dozens of cases achieved by endoscopic surgery for the treatment of TM caused by OLF and the application value of endoscopic surgery for this disease has received increasing attention and recognition [19–21]. In this study, the clinical results of 16 patients showed there were no complications, such as cerebrospinal fluid leakage, deep venous thrombosis of the lower extremities, nerve injury or incision infection. The mJOA score, VAS score and ODI score significantly improved from preoperative to postoperative 1 year. The overall Hirabayashi recovery rate was 73.96% ± 16.58% by calculating MJOA scores before operation and at the last follow-up, which is comparable to the reported rates of 60.5–65% in the literature [9, 22–24]. According to the Macnab efficacy evaluation, the excellent and good rate was 87.5%. Compared with traditional open surgery (34.6–76.7%) [8], this method may have obvious advantages. The above results serve as strong evidence of the clinical treatment effect of posterior full endoscopic surgery on TM secondary to OLF. Endoscopic treatment for thoracic OLF leads to a short hospitalization time, a small volume of intraoperative bleeding, small surgical incisions, rapid recovery, and few postoperative complications. During the operation, the intraspinal structure can be clearly observed, the location of the lesion can be identified, and the possibility of spinal cord, nerve and dural injury can be reduced, thus improving the safety of treatment. The original intent of this endoscopic procedure was to achieve the same results as open surgery for this disease with less trauma including skin incision size, preservation of bone structure, and damage to paravertebral tissues such as the paravertebral muscles. The follow-up of the current patients did not show any significant impact on the spinal structure. The clinical and imaging data show that the procedure leaves only 1–2 bone holes of less than 1 cm in diameter in the vertebral plate, and there is a tendency for bone healing after surgery, which had much less impact than open surgery. Compared with open surgery, this surgery can achieve full decompression without damaging paravertebral muscle tissues or reducing spinal stability. The OLF area and spinal canal invasive proportion of 1 year after the operation were compared with the values preoperatively. Five cases showed relatively significant exacerbating trends, indicating that after full endoscopic surgery, OLF still has the possibility of progressing further. This finding should be verified by longer-term follow-up data.

OLF preoperative imaging can help surgeons determine the shape, size and exact location of the OLF and help the surgeons formulate a suitable operation plan. There have been studies on imaging classification systems and evaluations of the degree of cervical and lumbar stenosis [25–28]. The occupied area and proportion of invasion into the spinal canal have been measured in individuals with diseases such as lumbar disc herniation. Some scholars have also proposed evaluating the residual area proportion of OLF compression in CT spinal canal cross-sections and the severity of ligamentum flavum ossification [29], the area can be accurately measured to assess the degree of spinal stenosis at a single level. In this study, the transected and sagittal ossification proportions of the ossified ligamentum flavum were used as the imaging evaluation standards. The proportion of ossification area of ligamentum flavum in sagittal position and transverse position three days and one year after surgery was significantly reduced compared with that before surgery, and there was no significant change one year after surgery compared with three days after surgery, suggesting that endoscopy has a significant effect on TOLF treatment Comparative analysis of the measured area data obtained by CT imaging can intuitively display the changes in the range of lesion sites before and after TOLF patients. The radiographic measurements and clinical scores (VAS, mJOA, ODI) in 16 patients are inversely related. In 16 cases, the ossification area and canal invasive proportion of the ligamentum flavum were measured accurately regarding the compression of the spinal canal in TM caused by OLF patients and there was a significant correlation between the improvement of symptoms and the decrease of proportions. Based on this finding, with accurate and standard high-density CT scanning and 3D reconstruction technology, it is expected that the occupied proportion of volume of the spinal canal can be used as an evaluation standard of thoracic myelopathy in the future.

Treatments for lumbar disc herniation, cervical disc herniation, cervical disc herniation, cervical nerve root canal stenosis and other degenerative diseases in our department have yielded good results, and the clinicians have accumulated a great deal of experience in endoscopic surgery. Because the location, shape and size of OLF lesions vary across individuals, the surgical decisions, surgical approach and overall design need to be determined carefully on the basis of all previous clinical experiences. Moreover, the learning curve for full endoscopic spinal surgery is steep. When applied to TM caused by OLF, because of the unique anatomical characteristics of the thoracic vertebrae, the operation is more difficult and riskier. Surgeons with sufficient experience in full endoscopic spinal surgery are needed.

There are some limitations to this study, including the following: the sample size was limited, and larger sample sizes are needed to assess OLF in the later stage; the follow-up time was short, and additional studies on the long-term efficacy, complications, etc. are needed; more studies on imaging measurements, studies aiming to determine whether endoscopic techniques are adequate for OLF resection and spinal canal decompression, and comparative studies on traditional surgical methods are needed.

## Conclusion

The endoscopic surgery for the treatment of TOLF has the advantages of less trauma to the paraspinal muscles and no impact on the spinal structures, which has a good clinical effect on TOLF. The CT-based radiographic measurements can quantitatively evaluate the degree of spinal canal stenosis in TOLF. Moreover, according to the specific conditions of the patients, we should make individualized diagnosis and treatment plans, integrate medical imaging with clinical medicine organically, and strive to develop more accurate and suitable diagnosis and treatment methods so that this disease can be prevented in the early stage of onset in the majority of patients.

## Data Availability

The datasets used during the current study are available from the corresponding author on reasonable request.

## Abbreviations

CT: Computed tomography
TM: thoracic myelopathy
OLF: ossification of the ligamentum flavum
mJOA: the modified Japanese Association of Orthopedics scale
VAS: visual analog scale for pain
ODI: Oswestry lumbar dysfunction index
